# Lipid-driven epigenetic exhaustion: linking palmitate metabolism to CD8^+^ T-cell dysfunction in cancer

**DOI:** 10.1097/IN9.0000000000000086

**Published:** 2026-07-22

**Authors:** Desh Raj, Mahesh Kathania, Tuoqi Wu, Arianne L. Theiss, K. Venuprasad

**Affiliations:** 1 Department of Internal Medicine, UT Southwestern Medical Center, Dallas, TX, USA; 2 Department of Immunology, UT Southwestern Medical Center, Dallas, TX, USA; 3 Harold C. Simmons Comprehensive Cancer Center, UT Southwestern Medical Center, Dallas, TX, USA; 4 Division of Gastroenterology and Hepatology, Department of Medicine, University of Colorado School of Medicine, Aurora, CO, USA

**Keywords:** T-cell exhaustion, cytotoxic T cells, palmitate, acetyl CoA

## Abstract

In a recent study published in *Science Immunology*, Tiberti and colleagues demonstrate that palmitate, a saturated fatty acid enriched in tumors, directly impairs CD8^+^ cytotoxic T lymphocyte function through mitochondrial and epigenetic reprogramming. Palmitate exposure reduced mitochondrial fitness, oxidative phosphorylation, and adenosine triphosphate production, resulting in defective proliferation, cytokine production, and antitumor activity. Mechanistically, mitochondrial dysfunction decreased intracellular acetyl-CoA availability, leading to reduced histone acetylation and loss of chromatin accessibility at loci that control effector programs. The study further identifies sphingosine kinase 2 as a critical mediator of lipid-induced dysfunction. Importantly, SPHK2 inhibition restored mitochondrial function, histone acetylation, and cytotoxic T lymphocyte antitumor activity, highlighting a potential therapeutic strategy for enhancing cancer immunotherapy.

The tumor microenvironment (TME) imposes profound metabolic constraints on infiltrating immune cells. Among these, lipid accumulation has emerged as a defining feature of many solid tumors, where excess fatty acids shape not only tumor cell behavior but also antitumor immunity ^[1]^. In their recent study published in *Science Immunology*, Tiberti and colleagues provide compelling evidence that palmitate (PA), a saturated fatty acid enriched in tumors, directly impairs CD8 T-cell function by inducing mitochondrial dysfunction and epigenetic repression through a sphingosine kinase 2 (SPHK2)-dependent pathway ^[2]^. Their work identifies a mechanistic connection among lipid metabolism, mitochondrial fitness, histone acetylation, and T-cell dysfunction, offering new insights into how excess nutrients contribute to immune evasion.

CD8^+^ cytotoxic T lymphocytes (CTLs) require extensive metabolic reprogramming to sustain activation, proliferation, and effector functions ^[3]^. Upon activation, T cells increase glycolysis and mitochondrial oxidative phosphorylation to meet biosynthetic and energetic demands ^[3]^. However, the nutrient landscape of tumors is profoundly abnormal ^[4]^. While glucose deprivation and hypoxia have been extensively studied as suppressors of T-cell activity ^[3]^, the role of excess lipids in regulating CTL fate remains less well understood. Tiberti et al ^[2]^. address this important gap by demonstrating that PA exposure alone is sufficient to induce a stable dysfunctional state in CTLs.

The study shows that PA-treated CTLs exhibit impaired activation, reduced proliferation, diminished cytokine production, and defective tumor-killing capacity ^[2]^. Importantly, these defects persist even after removal of PA, suggesting that exposure to saturated fatty acids induces durable cellular reprogramming rather than transient metabolic stress ^[2]^. This observation is particularly significant because it implies that T cells entering lipid-rich TMEs may acquire a long-lasting, dysfunctional phenotype that cannot be easily reversed by conventional activation signals.

A central conceptual advance of this work is the identification of mitochondrial dysfunction as the initiating event linking lipid overload to T-cell failure. PA exposure reduced mitochondrial mass, membrane potential, oxygen consumption, spare respiratory capacity, and adenosine triphosphate (ATP) production. These findings reinforce the growing recognition that mitochondrial fitness is indispensable for sustaining CTL effector function. Previous studies have established that dysfunctional mitochondria contribute to T-cell exhaustion during chronic infection and cancer ^[5]^. However, Tiberti and colleagues now place saturated fatty acid accumulation upstream of this process, suggesting that lipotoxicity itself may drive mitochondrial collapse in tumor-infiltrating T cells ^[2]^.

One of the most intriguing aspects of the study is the mechanistic connection between mitochondrial metabolism and epigenetic regulation. The authors demonstrate that PA-induced mitochondrial dysfunction reduces the intracellular availability of acetyl-coenzyme A, leading to decreased histone acetylation, particularly at H3K27ac and H3K9ac marks associated with active chromatin. This reduction in histone acetylation was associated with a widespread loss of chromatin accessibility and transcriptional silencing of genes involved in T-cell activation, proliferation, and cytotoxicity.

These findings highlight an emerging principle in immunology: cellular metabolism is not merely supportive of immune function but also directly instructs cell fate through epigenetic remodeling. In CTLs, acetyl-CoA serves as a metabolic substrate for histone acetylation, thereby linking mitochondrial activity to transcriptional competence. By demonstrating that lipid-induced mitochondrial dysfunction reshapes chromatin accessibility, the study provides an elegant example of how environmental metabolites can dictate immune cell identity at the epigenetic level.

The integration of RNA-seq and ATAC-seq further strengthens the study. PA-treated CTLs displayed repression of genes associated with cell-cycle progression and effector differentiation, including granzyme B, perforin, and interferon-g. Concurrently, chromatin accessibility decreased at loci enriched for AP-1 family transcription factor motifs, which are critical regulators of T-cell activation and effector programs. These data support a model in which PA exposure drives a broad transcriptional collapse that resembles features of dysfunctional or exhausted T cells. Beyond defining the metabolic and epigenetic consequences of PA exposure, Tiberti et al ^[2]^ identify SPHK2 as a key mediator of this dysfunctional state. Lipidomic analyses revealed that PA induces substantial remodeling of sphingolipid metabolism, including accumulation of ceramides and sphingolipid intermediates. SPHK2 expression was elevated in PA-exposed CTLs and in tumor-infiltrating CTLs from pancreatic cancer models. Pharmacologic inhibition or genetic deletion of SPHK2 restored mitochondrial fitness, histone acetylation, cytokine production, and tumor-killing capacity. These findings are particularly exciting because they identify a therapeutically actionable pathway. SPHK2 inhibitors are already under clinical investigation in oncology, and the present study suggests that their benefit may extend beyond direct tumor cell targeting to include restoration of antitumor immunity. In adoptive cell transfer models, SPHK2 inhibition rescued the functionality of PA-exposed CTLs and improved tumor control in vivo, underscoring the translational relevance of the work (Figure 1).

**Figure 1.**
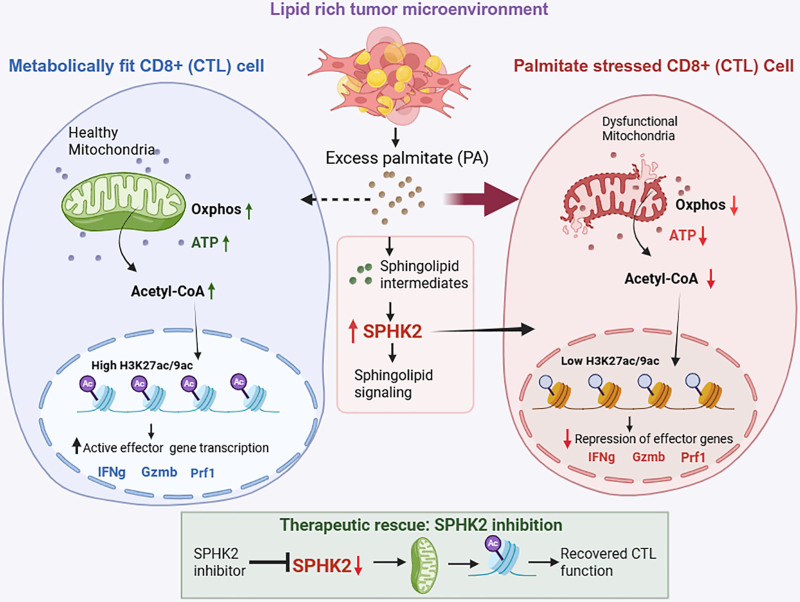
**Palmitate-driven mitochondrial and epigenetic dysfunction impairs antitumor CD8 T-cell immunity.** In lipid-rich tumor microenvironments, excess palmitate is taken up by CD8^+^ cytotoxic T lymphocytes (CTLs), triggering sphingolipid remodeling and upregulation of sphingosine kinase 2 (SPHK2). Increased sphingolipid and ceramide accumulation promotes mitochondrial dysfunction characterized by reduced mitochondrial membrane potential, impaired oxidative phosphorylation, and diminished adenosine triphosphate (ATP) production. This metabolic collapse decreases intracellular acetyl-coenzyme A availability, resulting in reduced histone acetylation and loss of chromatin accessibility at loci associated with effector and proliferative programs. Consequently, palmitate-exposed CTLs exhibit impaired activation, diminished cytokine production, defective cytotoxicity, and compromised antitumor activity. Pharmacologic inhibition of SPHK2 restores mitochondrial fitness, histone acetylation, and CTL effector function, thereby improving tumor control. The figure was created in Biorender.

The study also raises several important questions for future investigation. First, it remains unclear whether PA-induced dysfunction fully overlaps with canonical T-cell exhaustion or instead represents a distinct lipotoxic dysfunctional state. While features such as reduced cytokine production and impaired mitochondrial fitness are shared, the precise transcriptional and epigenetic relationship between these states warrants further examination. Second, the mechanisms by which SPHK2 regulates mitochondrial integrity remain incompletely defined. Although the data strongly support the role of sphingolipid accumulation in promoting metabolic stress, additional work will be needed to determine how ceramides, membrane remodeling, and mitochondrial signaling interact to drive T-cell dysfunction. Third, it will be important to determine whether similar mechanisms operate in other immune populations within the TME. Macrophages, natural killer cells, and regulatory T cells are also profoundly influenced by lipid metabolism, and PA-mediated metabolic reprogramming may broadly shape immune composition and function in tumors. Finally, these findings have implications beyond cancer. Obesity and metabolic syndrome are associated with chronic elevations in circulating saturated fatty acids and impaired immune responses. The possibility that lipid-driven epigenetic remodeling contributes to immune dysfunction in these settings deserves careful exploration.

Overall, Tiberti and colleagues provide a compelling framework linking lipid excess to immune suppression through mitochondrial and epigenetic reprogramming ^[2]^. Their work expands the current understanding of immunometabolism by identifying PA not simply as a nutrient source but as a potent regulator of T-cell fate. By connecting mitochondrial dysfunction to histone acetylation and chromatin accessibility, the study reveals how metabolic stress can become epigenetically encoded in antitumor T cells.

As the field increasingly recognizes metabolism as a determinant of immune cell identity, targeting pathways that preserve mitochondrial and epigenetic fitness may emerge as an important strategy to enhance cancer immunotherapy. The identification of SPHK2 as a metabolic checkpoint in lipid-stressed CTLs opens a promising avenue for therapeutic intervention and underscores the importance of accounting for the TME lipid landscape when designing next-generation immunotherapies.

## Conflicts of interests

The authors declare that they have no conflicts of interest.

## Funding

This work was supported by funds from the National Institutes of Health (R01-AI155786, R01-CA266072, and 1R01-CA282143) to KV.
